# Plastic and Reconstructive Surgery

**Published:** 1919-08-16

**Authors:** 


### WAR AND SURGICAL PROGRESS.

### V. Plastic and Reconstructive Surgery.

ALTHOUGH the æsthetic and personal side of the highly successful results obtained in plastic surgery during the late war has brought these matters very prominently before the public, we must remember that during the twenty-five years preceding hostilities there was no other branch of the art in which such remarkable advances had been made. Even if we take for granted the fact that first antisepsis and then asepsis made this progress possible with cases needing transplantation of bone or tissue, there still remains the certain knowledge that the ancients, as recorded in their histories, practised many and extensive plastic operations.

In recent times the ingenuity of the most skilful surgeons of the day was exerted to enable them to remedy cleft palate or hare lip, and a host of other congenital deformities; also they met the results of accidents, burns, and the deformities following lupus or malignant growth. Fragments, grafts, and flaps, taken from all parts of the body, have been utilised, and many now famous names were first associated with this work; but in spite of the breadth of this field for their activities, the horrors of war multiplied many times the number of patients requiring such treatment. Such restorative surgery includes, strictly speaking, work along several different lines—namely, bone grafting, tendon transplanting, restoring continuity with adipose or other tissue, etc. But perhaps the most striking, if not the most vital, results have been obtained in cases involving the jaws and the remedying of gross defects of the face and mouth. It is to this particular section that these notes are chiefly confined, for modern warfare has resulted in much deformity, particularly distressing when involving this region, and even though the fighting is now over it is to plastic surgery that many of these unfortunates are still looking for relief.

### THE CARE OF PLASTIC CASES.

Among the important responsibilities to be faced at present is the extensive after-care which must be given to those whose injuries require plastic work, for though tne early requirements are that immediate steps be taken to prevent contracture of muscles, stiffness of joints, and atrophy of tissues, it is obvious that plastic surgery must in a great many instances be applied periodically over a number of years. Just as organised care has been established for those men who suffered amputation, so should we make plans for the post-war treatment of these unfortunate plastic cases. To some extent this has been done, but one is probably justified in saying that a further extension of centres where these cases are readily understood and dealt with would be both deserved and invaluable. It is too late now to point out the once essential need that such patients should be brought back immediately to completely equipped hospitals and some temporary adjustments made before they undertake any journey the length of which may give time for hopeless contractions to occur, so that it now remains but to record for future guidance those new principles which experience has taught us.

### CO-OPERATION WITH THE DENTAL SURGEON.

Probably the most important of these is the recognition that there must be the closest co-operation between the general surgeon and the dental surgeon. Obviously in the organisation of all hospitals the latter is established as indispensable, and the value of his routine work cannot be overestimated. But the field of his activities has been greatly enlarged by reason of the vast number of jaw and face wounds encountered, resulting in loss of bone and teeth, as well as soft parts. It is another instance of successful team work, and the brilliant results obtained by those units which encouraged this co-operation leave no doubt as to its advisability.

### FRACTURES OF THE MANDIBLE.

Even when, by wiring, etc., the bony fragments have been worked into reasonable position, one of the most troublesome conditions (where soft parts are involved also) is failure in closing the jaws, and this may be due to one of two causes. The first, contraction of the scar tissue, may be treated by stretching with a variety of mechanical appliances, or by excision of the scars themselves. The latter method is said to be preferable for both permanency and cosmetic reasons. With regard to splinting, it is to be noted that if the mucous surfaces inside the mouth be broken, appropriate flanges must be inserted between the cheek and the jaw to prevent union of the raw surfaces. The second, obliteration of the buccal sulcus, is also best treated by dividing soft tissue when necessary, and again utilising splints with appropriate flanges to keep apart the soft structures until their surfaces have become epithelialised. It is a curious fact that even with extensive comminution it is rare for ankylosis of the temporomandibular joint to occur, and provided soft-part contracture be satisfactorily limited, and movements of jaw periodically kept up, then the more purely plastic operations can be hopefully undertaken.

### RE-FORMING LIPS AND CHEEKS.

One difficulty encountered is the production of flaps of mucous membrane to line the cutaneous flaps, and for this purpose Cole has successfully used skin areas previously and, in his case, permanently depilated by radiation. For the full and most interesting description of the method one must refer to this surgeon's own writings. Gillies has used double-bridge flaps brought down from the scalp to replace lost skin over the chin, and also inserted a large osteo-cartilagenous graft from the seventh rib, which gave considerable power of mastication where previously there was none, and this instance illus-

☛ Previous articles appeared on May 24, June 7 and 28, July 19, p. 405.

**War and Surgical Progress**—(*continued*).

trates the fact that though the skin of the chin and cheeks can be replaced fairly readily by simple flaps and skin grafts, yet if any extensive loss of bone has occurred the only satisfactory way to regain real biting power for the jaw is to apply a direct bone graft.

### Bone Grafts to Jaws.

Several surgeons have described cases of successful free bone grafts. Platt, Campion, and Rodways, in 1918, gave nine instances, and their notes are also of interest as pointing out possible causes of failure. Bony union may not occur with a functionless ascending ramus, for if operation be delayed the bone may become atrophied and of low vascularity. Also these surgeons did not fix their grafts by wires, but made a bone flap at one end of the fragments of the mandible, and forced the ends of the graft into this for support, afterwards suturing the wound in successive layers. Cole, unless he can fix the graft by similar wedging, follows the technique of Lane and Hey Groves, using metal plates to attach the ends, but apparently septic complications were not uncommon. More successful was this surgeon in taking pedicled grafts from the lower border of the mandible to bridge across a gap in the same bone. His method, fully described in the *British Journal of Surgery*, was briefly as follows: A suitable splint is first applied, and by a skin incision the anterior fragment was fully exposed, but only the extremity of the proterior. The former was sawn through at its lower point, the periosteum on the buccal surface divided, and a pedicle defined by incisions through the platysma and deep fascia. The graft was drawn into position and secured by silver wires to both fragments. A drainage tube was inserted for twenty-four hours, and then the wound completely closed. In this way a graft 3.5 cm. long has been obtained.

In France Delagenière has been most successful with direct grafts taken from the tibia, and although not applied to face injuries alone, the following description, as given by the *American Naval Bulletin*, is of interest:

The fresh osteoperiosteal grafts from the tibia are immediately transferred to the operation wound without intermediaries of any kind, taking care to handle them only with sterile compresses or instruments. The tibia is treated simply by rapid skin suture over the denuded bone surface, from which the periosteum has been stripped, and a small drain is left under the skin for forty-eight hours in order to guard against the formation of a hæmatoma; the wound heals in eight to ten days without complications. The graft must be transferred without delay to its new position in the interest of perfect asepsis. The employment of antiseptic agents is contra-indicated as interfering with the vitality of the graft. As far as possible, the two surfaces of the graft must be in contact with living tissues. While this condition is easily met with in the closing of bony defects of the skull, more serious difficulties are encountered in the case of extremities. The entire graft must be well covered with skin in order to guard against necroses and sequestration of the uncovered bony portions of the grafts. . . . Conditions in the facial region are especially favourable for the handling of grafts, which can be placed in living tissues where it is easy to avoid dead spaces and to secure good hæmostasis. Failure is accordingly rare and almost invariably due to the opening of a natural cavity of the face. The bony framework of the nose can be entirely repaired by means of these osteoperiosteal grafts.

In certain cases, however, the injury may be too great to effect any satisfactory repair, and this is most frequently the case with injuries to the superior maxillæ.

### Injuries to the Maxillæ.

If the soft tissue and bony loss be small it is comparatively easily replaced by a mechanical appliance, but when large, terrible facial deformity may be produced, and here arise the supreme test of the combined efficiency of the plastic surgeon and the mechanical adjustable denture. It is impossible, with the space at our disposal, to even outline the finer details. The principles already mentioned hold good, and their successful application is more than proved by the number of unfortunate men whose countenance had lost all semblance to humanity through the frightful ravages of war, but who have now regained not only some semblance of their former looks, but a high degree of mobility and local muscular efficiency. This war-work must be considered as one of the greatest triumphs achieved by the art of surgery.

